# Superconducting Quantum Interferometers for Nondestructive Evaluation

**DOI:** 10.3390/s17122798

**Published:** 2017-12-06

**Authors:** M. I. Faley, E. A. Kostyurina, K. V. Kalashnikov, Yu. V. Maslennikov, V. P. Koshelets, R. E. Dunin-Borkowski

**Affiliations:** 1 Peter Grünberg Institute, Forschungszentrum Jülich GmbH, 52428 Jülich, Germany; r.dunin-borkowski@fz-juelich.de; 2 Moscow Institute of Physics and Technology, Moscow 141700, Russia; kostyurina.katya@gmail.com (E.A.K.); kalashnikovkv@gmail.com (K.V.K.); 3 Kotel’nikov Institute of Radio Engineering & Electronics RAS, Moscow 125009, Russia; cryoton@inbox.ru (Y.V.M.); valery@hitech.cplire.ru (V.P.K.)

**Keywords:** magnetic analysis, magnetic sensors, nondestructive testing, scanning probe microscopy, SQUIDs

## Abstract

We review stationary and mobile systems that are used for the nondestructive evaluation of room temperature objects and are based on superconducting quantum interference devices (SQUIDs). The systems are optimized for samples whose dimensions are between 10 micrometers and several meters. Stray magnetic fields from small samples (10 µm–10 cm) are studied using a SQUID microscope equipped with a magnetic flux antenna, which is fed through the walls of liquid nitrogen cryostat and a hole in the SQUID’s pick-up loop and returned sidewards from the SQUID back to the sample. The SQUID microscope does not disturb the magnetization of the sample during image recording due to the decoupling of the magnetic flux antenna from the modulation and feedback coil. For larger samples, we use a hand-held mobile liquid nitrogen minicryostat with a first order planar gradiometric SQUID sensor. Low-T_c_ DC SQUID systems that are designed for NDE measurements of bio-objects are able to operate with sufficient resolution in a magnetically unshielded environment. High-T_c_ DC SQUID magnetometers that are operated in a magnetic shield demonstrate a magnetic field resolution of ~4 fT/√Hz at 77 K. This sensitivity is improved to ~2 fT/√Hz at 77 K by using a soft magnetic flux antenna.

## 1. Introduction

Nondestructive evaluation (NDE) describes the characterization of the structure and/or functionality of an object without compromising its usability. The recording of magnetic fields is a non-invasive contactless method that provides a direct view of magnetic features and/or electrical currents deep in the object. For an NDE technique that involves magnetic field measurement, it is challenging to construct a magnetic sensor that has high magnetic field sensitivity, high dynamic range and a broad frequency bandwidth that allows high sampling rates. Superconducting quantum interference devices (SQUIDs) provide unprecedented sensitivity down to the sub-fT/√Hz range, a broad frequency range of >1 MHz and a dynamic range of up to ~120 dB [1]. SQUID-based NDE systems have been developed for the investigation of objects that have dimensions of nanometers (nanoSQUID microscopes [2]) to kilometers (nondestructive archeology or geomagnetic evaluation [3,4]). Related scanning methods vary from 3D piezo stages to airborne systems transported by planes or helicopters. Successful applications of SQUID-based NDE systems from the last thirty years for monitoring materials and structures have been described and assessed elsewhere [5]. The disadvantages of such systems include their operation at cryogenic temperatures and, hence, the expense of performing routine measurements. In spite of the relatively high price of cryogenic equipment and technical difficulties, SQUID-based systems are employed when the required efficiency cannot be reached using alternative NDE techniques [6]. SQUID-based NDE systems have been developed and employed for the detection of defects in steel plates [7], the study of stress–strain states in ferromagnetic materials [8], the detection of ruptures in steel ropes on bridge structures [9], and the detection of cracks in turbine blades of aircraft engine turbine blades [10]. Here, we briefly review stationary and mobile low-T_c_ and high-T_c_ SQUID systems that have been developed in Forschungszentrum Jülich (FZJ) and the Kotel’nikov Institute of Radio Engineering and Electronics (IRE) for the NDE of room temperature objects, in the context of those developed elsewhere.

## 2. Basic Principle of Operation and Important Features of SQUIDs

A direct current SQUID (DC SQUID) is essentially a loop of superconductor interrupted by two Josephson junctions (JJs) that have non-hysteretic current-voltage characteristics and, in an ideal case, identical critical currents *I_c_* and normal state resistances *R_n_* (see [1,11,12] and references therein). The operation of SQUIDs is based on the dependence of the phase shift of the wave-function of Cooper pairs on the magnetic flux passing through the SQUID loop, similar to the phase shift of the wave-function of a charged particle in the Aharonov-Bohm effect. Both effects result from the fundamental dependence of the canonical momentum of a charged particle $\vec{p}=m\vec{v}+q\vec{A}$ on the magnetic vector potential $\vec{A}$ and represent a particular case of the presence of a geometric phase shift (Berry phase) in the wave function of a charged particle after its adiabatic evolution around a closed path in the parameter space of magnetic vector potentials [13]. A DC SQUID is sensitive to the magnetic flux *Ф* that passes through its loop, leading to spatial variations in the phase of the wave function of Cooper pairs in the superconducting electrodes. These spatial variations lead to phase shifts ∆ϕ_1_ and ∆ϕ_2_ at the Josephson junctions and, as a result, to a voltage signal. At an optimal bias current of *I_B_ ≅* 2*I_c_*, the DC voltage *V* on a DC SQUID depends periodically on the magnetic flux *Ф* that passes through the SQUID loop according to the expression [14]

(1) $$V\approx \frac{R_{n}I_{B}}{2}\sqrt{1-{\left(\frac{2I_{c}}{I_{B}}\cos\frac{\pi \Phi }{\Phi_{0}}\right)}^{2}},$$

where the modulation period is equal to the magnetic flux quantum *Ф*_0_ ≈ 2.07 × 10^−15^ T·m^2^. The periodic dependence of the SQUID voltage on magnetic field can be linearized by implementing a dynamic range higher than 120 dB and a slew rate larger than 1 M*Ф*_0_/s using the DC SQUID control electronics, providing a digital negative feedback signal within each period and counting the periods when the magnetic flux exceeds *Ф*_0_ [15].

According to Equation (1), a SQUID is sensitive to the magnetic flux *Ф* that penetrates through its loop. For sensitive measurements of magnetic fields, the SQUID should be equipped with a superconducting flux transformer that collects the magnetic flux in a pickup loop from a relatively large area and concentrates it into the SQUID loop using a multiturn input coil. The magnetic field sensitivity *B_N_* of a DC SQUID magnetometer with an inductively coupled superconducting flux transformer can be estimated according to the equation

(2) $$B_{N}=\frac{L_{pu}+L_{i}}{kA_{pu}\sqrt{L_{i}L_{S}}}S_{\Phi }^{1/2},$$

where *S_Ф_* is the magnetic flux noise of the high-T_c_ DC SQUID, *L_pu_* and *A_pu_* are the inductance and the area of the pickup loop, respectively, *k* is the coupling coefficient between the input coil and the SQUID loop, *L_i_* is the inductance of the input coil and *L_S_* is the inductance of the SQUID loop.

## 3. Low-T_c_ vs. High-T_c_ JJs and DC SQUIDs: Technologies and Properties

**Figure 1:**
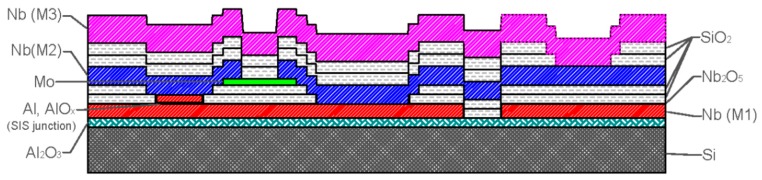
Schematic representation of a Nb-based low-T_c_ Josephson junction developed at IRE.

Currently, the most sensitive detector for subtle magnetic field measurements is a DC SQUID magnetometer based on low-T_c_ superconducting polycrystalline Nb films and planar JJs. A magnetic field resolution below 1 fT/√Hz at 4.2 K has been demonstrated [16]. Thin film JJs based on Nb films are widely implemented in superconducting electronics, including low-T_c_ DC SQUID magnetometers. The noise and signal characteristics of such magnetometers depend directly on the quality of the JJs. High quality JJs with a small spread of parameters over the substrate and between batches are vitally important for the development of low-noise sensors that are suitable for NDE applications. Several methods for the fabrication of shunted JJs have been developed. These methods include the use of double-barrier junctions with an additional normal layer between two conventional JJs [17,18] and Nb/αSi/Nb structures with a doped Si layer [19]. However, the most widely used and best-developed method involves the use of Nb/Al-AlO_x_/Nb tunnel junctions [20,21] with an additional external resistive shunt made from Mo (Figure 1). The Mo shunt resistor is highlighted in green in Figure 1.

One of the factors that results in a reduction in the quality of Nb-based junctions is the presence of internal mechanical stress in the thin superconducting Nb films, which can lead to destruction of the tunnel barrier and junction degradation. The surface roughness of the bottom electrode caused by the internal stress increases Al diffusion at the Nb-Al boundary and can lead to micro-shortcuts. These micro-shortcuts typically result in increased noise levels of the JJs and SQUIDs. In order to minimize tension in Nb films prepared using DC magnetron sputtering, the operating modes of the magnetron have been investigated. Experimental studies of the dependence of internal tension on magnetron power level and Ar pressure have shown that the optimal deposition of Nb films is realized at a power of ~600 W for a target area of ~122 cm^2^ and an Ar pressure of ~10^−2^ mbar.

**Figure 2:**
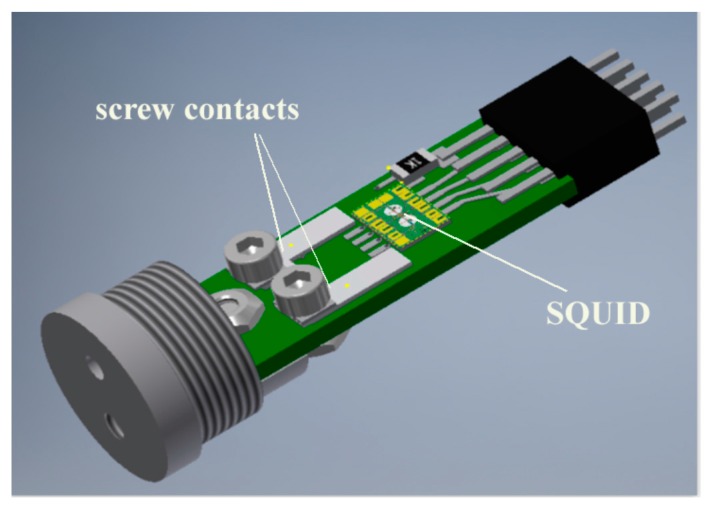
Schematic representation of Nb-based low-T_c_ DC SQUID sensor developed at IRE. The cylindrical superconducting (Nb) shield has been removed for clarity.

The typical capacitance of the Nb/AlO_x_/Nb JJs that are used in SQUID sensors is ~0.5 pF at a critical current density of the JJs of ~200 A/cm^2^ and an area of 3.2 µm × 3.2 µm [22]. Up to ~100 low-T_c_ DC SQUID structures with integrated input coils can be produced simultaneously on a single large-area Si wafer. Pick-up loops of superconducting flux transformers made from thin Nb wires can be used to measure the magnetic field or field gradient and to transfer it, in the form of an induced superconducting current, into the multiturn thin film input coil, which concentrates the magnetic flux into the SQUID loop, which is integrated on the same substrate. The SQUID sensor is placed in a superconducting shield, in order to isolate it from the parasitic influence of external electromagnetic interference. Standard highly sensitive low-T_c_ SQUIDs are available from commercial companies (see, for example, [23]). Special SQUID sensors that are intended for NDE experiments have been developed and produced in small quantities at IRE (see Figure 2). The primary advantage of using such self-made low-T_c_ SQUID sensors is the possibility to adapt their design to a particular NDE system, in order to reduce the coupling of parasitic background signals to the SQUID. The current design of a SQUID loop includes 4 balanced slots that are coupled gradiometrically to two input coils, one modulation coil and one feedback coil. The sensors are encapsulated inside a Nb shield together with screw contacts that are machined from Nb and provide a superconducting connection to the Nb wire of the gradiometric pick-up loops. The Nb contact pads on the SQUID chip are connected to the Nb screw contacts using a 25-µm-diameter Nb wire.

**Figure 3:**
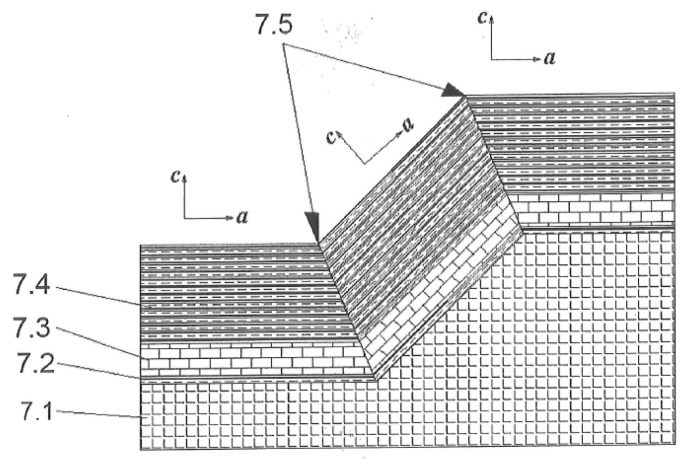
Schematic representation of a step-edge high-T_c_ Josephson junction developed at FZJ [28,29,30]. (7.1) Textured MgO substrate with a step height of ~400 nm; (7.2, 7.3) Graphoepitaxial buffer layers; (7.4) YBCO film; (7.5) Grain boundaries.

High-T_c_ JJs and SQUIDs are based on epitaxial films of the high-T_c_ superconductor YBa_2_Cu_3_O_7−x_ (YBCO). The much shorter and highly anisotropic coherence length in YBCO (ξ_ab_ ≈ 2 nm, ξ_c_ ≈ 0.4 nm), as well as the d-wave symmetry of the superconducting order parameter and the strong dependence of the order parameter on the local strain and oxygen content in YBCO, when compared to the isotropic coherence length ξ ≈ 38 nm and s-wave symmetry of the superconducting order parameter in polycrystalline Nb films, results in a completely different technology for high-T_c_ JJs. Grain boundaries can play the role of weak links in YBCO, whereas they do not significantly suppress the superconducting order parameter in Nb. High-T_c_ JJs are based mainly on grain boundary weak links, which can be realized by the epitaxial growth of YBCO films on bicrystal substrates [24,25] or on sharp steps etched on the surfaces of single crystal substrates [26,27,28,29,30,31]. Step-edge JJs can be placed on any part of a substrate, allowing the more efficient use of the substrate surface to design more efficient SQUID structure(s) with grain boundaries that are located exclusively at the JJ (see Figure 3). Newly-developed high-T_c_ step-edge JJs are based on the presence of two synchronously operating 45° [100]-tilted grain boundaries and possess optimal parameters for operation in high-T_c_ DC SQUIDs: critical current *I_c_* ≈ 40 µA, capacitance *C* ≈ 10 fF, normal state resistance *R_n_* ≈ 20 Ω and characteristic voltage *I_c_R_n_* ≈ 800 µV at 77 K [28,29,30,31]. The 50 times smaller capacitance of high-T_c_ JJs when compared to the capacitance of low-T_c_ JJs is advantageous for the low noise properties of high-T_c_ DC SQUIDs based on high-T_c_ JJs. In comparison to high-T_c_ step-edge JJs on SrTiO_3_ (STO) and LaAlO_3_ (LAO) substrates, such buffered 45° [100]-tilted step edge JJs on MgO substrates demonstrate better reproducibility and have lower noise values, also because of the absence of multiple low-angle grain boundaries at the bottom corner of the step.

Only a few high-T_c_ SQUIDs can be produced simultaneously on the relatively small single crystal substrates of STO, LAO and MgO materials that are used for deposition of the epitaxial high-T_c_ films and heterostructures. The sensitivity of a high-T_c_ SQUID is typically improved by using a thin film pick-up loop that is connected directly to the SQUID loop or inductively coupled to it via a multiturn input coil. Low noise high-T_c_ superconducting flux transformers are made from epitaxial films because of the absence of sufficiently flexible and thin high-T_c_ superconducting wires. Thin film 20-mm multilayer superconducting flux transformers based on heterostructures with YBCO films are used to concentrate magnetic flux into the loop of the high-T_c_ SQUID to achieve a magnetic field resolution of ~4 fT/√Hz at 77 K [25,31]. Further improvements in the magnetic field resolution of flip-chip high-T_c_ SQUID magnetometers down to ~2 fT/√Hz at 77 K have recently been achieved by using a soft magnetic flux antenna in addition to the 20-mm multilayer superconducting flux transformer [32].

High-T_c_ SQUIDs demonstrate low noise properties up to temperatures of ~80 K, which can easily be reached by cooling using relatively cheap liquid nitrogen or energy-efficient cryocoolers. A wide variety of high-T_c_ SQUID sensors have been developed for specific NDE applications. Typically, they are vacuum-tight-encapsulated in fiberglass capsules together with a heater and feedback coil. The propensity of YBCO films and MgO substrates to degrade in the presence of humidity or corrosive contaminants in the air results in the need for vacuum-tight encapsulation or passivation, which is required for long-term stability of the high-T_c_ SQUID sensors.

## 4. Low-T_c_ and High-T_c_ SQUID NDE Systems

A wide variety of NDE systems equipped with specific SQUID sensors have been developed to study objects with different requirements. The measurement of magnetic fields generated by remote objects in magnetically unshielded environments during nondestructive archeological or geomagnetic surveys can be performed to a first approximation using room temperature magnetometers such as fluxgates, induction coils or optically pumped magnetometers. A low-T_c_ SQUID gives the best results for apparent resistivity at both shallow and deep regions simultaneously because it covers a larger response time interval than conventional coils during transient electromagnetic measurements [33], which require frequency-independent sensitivity at the level of several fT/√Hz. During transient electromagnetic measurements, electromagnetic fields are induced by transient pulses of electric current through a large loop of wire and the subsequent decay response from currents induced in underground layers can be measured. As a result of their superior sensitivity at low frequencies, only SQUID systems are currently able to resolve changes in the electrical conductivity of underground layers with sufficient sensitivity for depths exceeding ~500 m. Both low-T_c_ and high-T_c_ mobile systems have been demonstrated for the recording of magnetic anomalies during movement of the systems in the Earth’s magnetic field [3,33,34,35]. High-T_c_ SQUID magnetometers or gradiometers with directly coupled 8-mm pick-up loops that are inductively coupled to first-order single-layer superconducting gradiometers, as well as low-T_c_ SQUID gradiometers with integrated multilayer gradiometric flux transformers [22], are currently the most suitable low-T_c_ SQUID sensors for mobile geomagnetic and archeological NDE.

The nondestructive monitoring of ion beam currents in particle accelerators is performed by the non-invasive measurement of magnetic fields generated by moving charged elementary particles. By using a Cryogenic Current Comparator (CCC) based on a low-T_c_ SQUID with a ferromagnetic Vitrovac core in the pick-up loop, a resolution of ~6 pA/√Hz at 4.2 K and 2 kHz with a system 10-kHz frequency bandwidth has been achieved for monitoring accelerated electrons or ^20^Ne^10+^ ions [36]. The sensor part of the CCC was optimized for the lowest possible noise-limited current resolution, in combination with a high system bandwidth of ~200 kHz, without compromising the resolution [37]. The ferromagnetic core was made from NANOPERM^®^ with different annealing recipes by the company MAGNETEC. The fine structure of a beam could be observed. The CCC could also be used for the calibration of different devices, such as a secondary electron monitor. By using a ferromagnetic-core-free monitor based on a high-T_c_ DC SQUID gradiometer with a multilayer flux transformer operating at 77 K, fabricated at FZJ, the intensity of a 1 μA beam of ^132^Xe^20+^ (50 MeV/u) ions could be measured non-invasively with 100 nA resolution [38].

In “traditional” NDE, high-T_c_ DC SQUID systems have demonstrated their superior capabilities for the inspection of metal plates, aircraft wheels and fuselage and pre-stressed concrete bridges [5,6,39,40,41,42]. The chosen measurement scheme depends on the NDE application: an eddy current excitation scheme and a narrowband lock-in readout scheme are used for the investigation of metal plates and aircraft parts, while measurements of static magnetic fields are efficient for monitoring magnetic flux leakage from ferromagnetic objects such as the pre-stressed steel tendons of concrete bridges. Deeper defects can be detected using SQUIDs at lower excitation frequencies, when compared to the conventional eddy current technique based on induction coils, because the sensitivity of coils decreases strongly with frequency.

**Figure 4:**
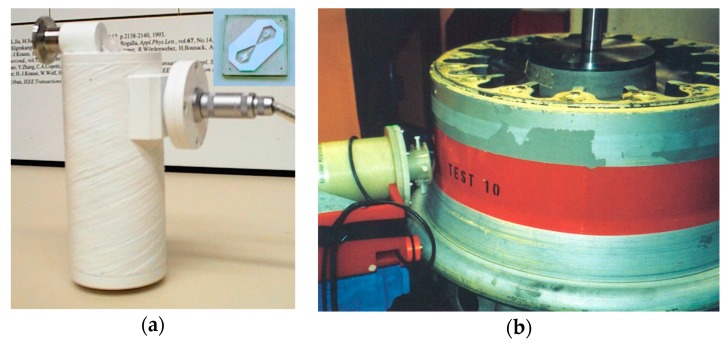
(**a**) Liquid nitrogen minicryostat used for the operation of a high-T_c_ DC SQUID gradiometer in an NDE system. The inset shows a photograph of the directly coupled high-T_c_ DC SQUID first order planar gradiometer, which was produced on a 1 cm^2^ LAO substrate and installed in the cryostat; (**b**) Scan of an airplane wheel rim using the high-T_c_ DC SQUID gradiometer system. The robotic arm scanner moves the cryostat along the outer surface of the wheel rim, while the wheel is rotated around its axis.

Figure 4a shows a nonmagnetic ~200 mL cryostat with fiberglass walls that is able to hold liquid nitrogen for up to ~4 h while operating in different orientations (see Figure 4b). It was held by hand or fixed on the robotic arm of an automatic scanner during NDE measurements. A high-T_c_ DC SQUID first order planar gradiometer produced on a 1 cm^2^ LAO substrate with a [110] orientation of its edges was fixed on a sapphire rod in the vacuum part of the cryostat, which was cooled by liquid nitrogen and placed ~1 mm from the outer surface of the bottom of the cryostat. Such gradiometers are able to operate in industrial environments, while providing a high sensitivity of ~50 fT/cm√Hz at 77 K to the magnetic field gradient ∂B_z_/∂x.

An interesting application of high-T_c_ SQUIDs for the NDE of non-magnetic Al pipes involves the use of a magnetostrictive transmitter and sensor based on the use of pre-magnetized thin Ni plates to generate ultrasonic waves in the pipes and to convert the ultrasonic waves that are reflected from defects into magnetic signals, which can be measured contactlessly using a high-T_c_ SQUID gradiometer [43,44]. Another prospective application of high-T_c_ SQUIDs is a multi-channel system intended for the detection of magnetic metallic contaminants in packaged food [45].

**Figure 5:**
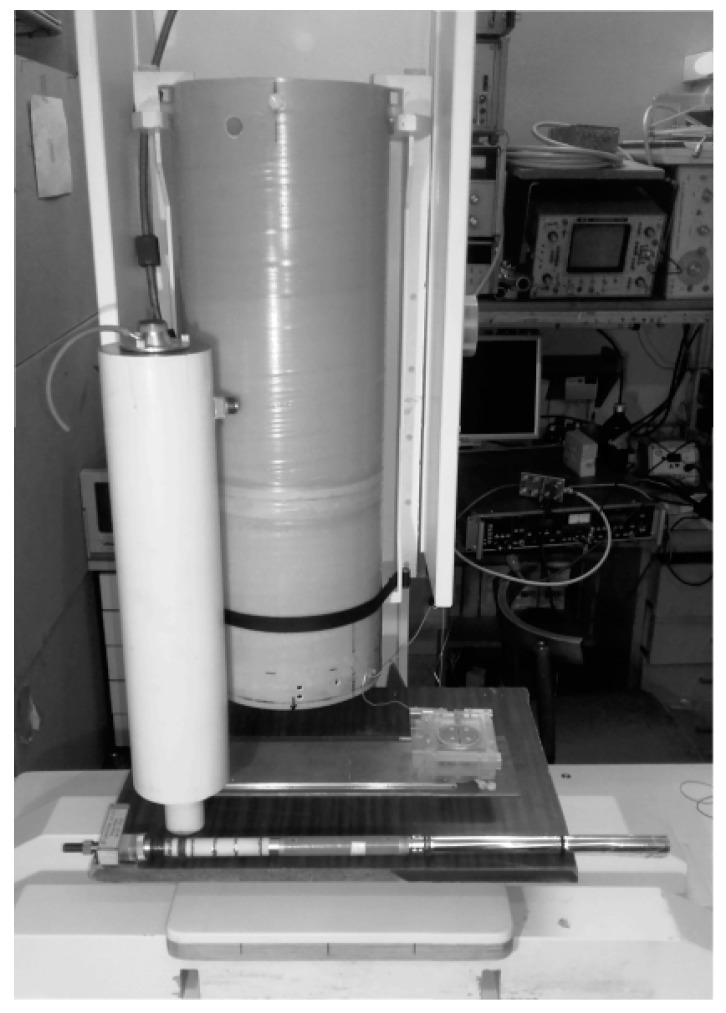
Photograph of a single-channel low-T_c_ DC SQUID-based gradiometer system with a liquid He cryostat and a measurement probe. The first-order gradiometer was made of insulated Nb wire with a diameter of 0.05 mm using a “1:1” configuration (one lower and one upper turn) on a textolite rod. The diameter of the pick-up loops of the gradiometer is 4 mm and the base line of the gradiometer is 40 mm. The initial unbalance of the gradiometer is below 1%. The gradiometer ends are fixed mechanically on the Nb lamella of the SQUID sensor for connection to the SQUID input coil.

At IRE, a low-T_c_ DC SQUID-based NDE system for operation in a magnetically unshielded environment was developed. The measurement probe in this system is based on fiberglass tubes and consists of the following elements: a first-order axial gradiometer as an input magnetic flux transformer, the low-T_c_ DC SQUID sensor CE2blue (a product of Supracon AG) with a low-T_c_ DC SQUID and input coil, connecting wires with a LEMO connector and a filling port for liquid He (see Figure 5). Low-T_c_ DC SQUID sensors developed at IRE are intended for the replacement of commercial sensors in future NDE systems.

The single-channel low-T_c_ NDE system includes a liquid He cryostat, as shown in Figure 5. The inner diameter of the neck and inner tail of the cryostat is 22 mm. The distance between the outer and inner surfaces in the tail in the cooled system is no greater than 10 mm. The working time of the cryostat, which is cooled by 1.2 L of liquid He, is more than 2 days. The parameters of the liquid helium cryostat are as follows: outer diameter 110 mm; length 500 mm; outer diameter of the tail 45 mm; outer length of the tail 85 mm; inner diameter of the neck 22 mm; inner diameter of the cryogenic volume 80 mm; weight of the empty cryostat 2.2 kg. As the cryostat volume is relatively small, the filling procedure is relatively simple and takes several minutes. The small volume of He and the presence of a relief valve result in safety of the cryostat if the vacuum conditions in the space between the inner and outer walls are violated.

In tests of the gradiometer in such a configuration, the transfer coefficient of the input magnetic field *В_in_* into magnetic flux *Ф_e_* in the SQUID was measured to be ~9.5 nT/*Ф*_0_, corresponding to an equivalent sensitivity of the gradiometer with respect to the magnetic field of ~30 fT/√Hz at a SQUID intrinsic noise level of 3 μ*Ф*_0_/√Hz. Such a sensitivity is sufficient for applications of SQUID-based gradiometers in NDE systems.

The DC SQUID electronics of the NDE system prototype are mounted on an Al box of size 117 mm × 62 mm × 19 mm located close to the cryostat and connected to the measurement probe using a cable of length 70 cm. The low-T_c_ DC SQUID electronics contain analog and digital components. The analog part contains a conventional modulation circuit of a null detector and a circuit of negative feedback with respect to magnetic flux. The analog components allow tuning of the low-T_c_ DC SQUID operating parameters. The digital components make it possible to switch the tuning and working regimes of the low-T_c_ DC SQUID gradiometer and system control using a personal computer. The low-T_c_ DC SQUID electronics are connected to the control unit by a 5-m-long cable. The preamplifier of the electronics unit is based on a Toshiba K-369 low-noise field effect transistor (FET) in the cascade circuit. The intrinsic noise of the preamplifier, without a transformer between the SQUID and the transistor, is <0.7 nV/√Hz. The transformer improves this value by approximately a factor of 10.

A single-pole integrator generates a feedback signal, which is fed to the modulation coil via a feedback resistor. The voltage across the feedback resistor is used as the output signal of the gradiometer. The DC SQUID electronics operate at a fixed feedback coefficient of ~1 V/*Ф*_0_. The bandwidth of the system is approximately 0–16 kHz. The control unit of the NDE system contains stabilized power supply sources and a data acquisition system based on a 24-bit ADC.

The elements described above were used to construct a working prototype of a DC SQUID-based gradiometer. The prototype was tested under laboratory conditions without additional magnetic shielding and the main working parameters were studied. The Stanford Research low-frequency spectrum analyzer was used to study the noise characteristics of the output signal of the DC SQUID-based gradiometer prototype.

Noise spectra were registered over a frequency interval of 1–1000 Hz at a feedback coefficient of К_FB_ = 1 V/*Ф*_0_. The measured transfer coefficient of the external magnetic field into the magnetic flux in the SQUID of ~9.5 nT/*Ф*_0_ corresponds to an equivalent noise level with respect to the magnetic field of ~30 fT/√Hz. Such noise levels of the DC SQUID-based gradiometer indicate sufficient balancing and confirm that such devices can be employed in NDE systems. DC SQUID-based gradiometers can be used to develop multichannel DC SQUID-based systems. The prototype of the single-channel DC SQUID-based gradiometer shows stable operation in unshielded laboratory conditions and can be used for the development of multichannel gradiometric DC SQUID-based systems for the NDE of defects in metal structures and materials.

One of the important elements of a SQUID-based NDE system is the XY-scanner used to scan samples under a stationary liquid helium cryostat. The developed XY-scanner was equipped with two computer-controlled stepper motors (5RK60GE-CW2TE, ORIENTAL MOTOR), in order to move samples in the X and Y directions. The scanned area was 300 × 300 mm, with an accuracy for sample positioning of ~0.3 mm. In order to avoid external magnetic noise from magnetic components, the sample holder was fabricated using non-metallic and non-magnetic materials, such as fiberglass and plexiglass.

## 5. High-T_c_ SQUID Microscope System with a Ferromagnetic Flux Antenna for NDE

**Figure 6:**
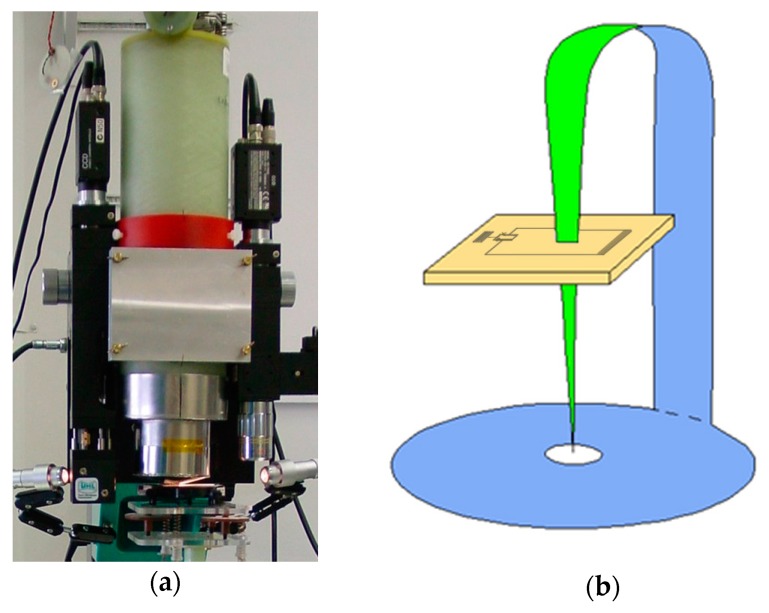
(**a**) Photograph of a high-T_c_ DC SQUID microscope with a fiberglass cryostat that can support 0.8 L of liquid nitrogen; (**b**) Schematic diagram of a high-T_c_ DC SQUID with a magnetic flux antenna made of soft magnetic foil penetrating the directly coupled pick-up loop [49].

A scanning SQUID microscope (SSM) is a powerful noninvasive tool for fundamental and applied research (see for example [2,46,47] and references therein). The high-T_c_ DC SQUID microscope developed at FZJ for studies of room temperature objects is based on a high-T_c_ DC SQUID with a magnetic flux antenna and was described in detail in our previous publications [48,49,50] (see Figure 6). Here, we review it briefly, report new results obtained with the system and provide an outlook for further developments.

The principle of operation of the microscope is shown in Figure 6b. An amorphous metallic soft magnetic 25 µm thick foil Vitrovac 6025X (Vacuumschmelze GmbH, Hanau, Germany) was used to guide magnetic flux from an object at room temperature through the pick-up loop of the high-T_c_ SQUID and to return the flux back to the object. 2-mm-wide stripes were cut using scissors in a direction normal to the rolling direction of the foil, in order to reduce Barkhausen noise from the ferromagnetic foil. The tip of the flux antenna was first formed at a 50° angle using scissors and the end of the tip was then sharpened to a radius of ~200 nm using 0.3 µm diamond polishing sheets.

**Figure 7:**
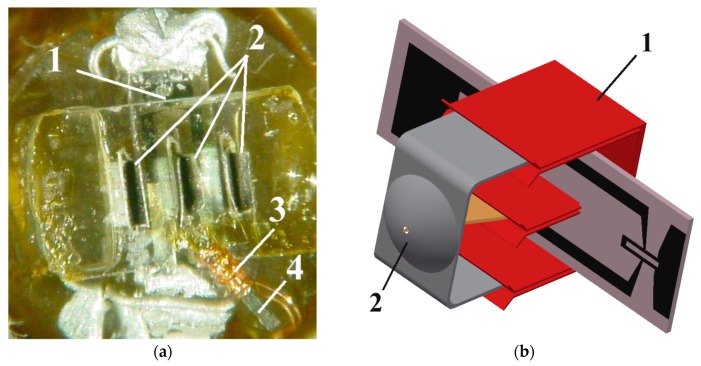
(**a**) Photograph of a high-T_c_ DC SQUID (1) assembled on a sapphire rod, showing parts of the magnetic flux antenna (2) and the modulation coil (3) on ferromagnetic wires (4); (**b**) Sketch of a DC SQUID with a directly coupled pick-up loop assembled together with low temperature (1) and room temperature (2) parts of the flux antenna.

The SQUID was fixed using vacuum grease on a sapphire rod together with the modulation coil and the low temperature part of the flux antenna (see Figure 7a) in the vacuum part of the cryostat. The sapphire rod was cooled using liquid nitrogen through the inner wall of the fiberglass cryostat. The cryostat contains ~0.8 L of liquid nitrogen when it is completely filled and provides 2 days of SQUID operation at a temperature of ~78 K. The room temperature parts of the flux antenna were vacuum-sealed using epoxy in the outer wall of the cryostat and connected to their cooled counterparts (see Figure 7b). Commercial ac-bias electronics was used for SQUID operation in flux-locked loop mode (Cryoton Co. Ltd., Moscow, Russia).

This system was used to perform measurements of the magnetic field distribution over a US $1 bill, for a qualitative comparison of the device with SQUID microscope systems made by other groups [51,52]. The magnetic signal originates from the black ink used for printing banknotes, which contains a small quantity of magnetite (Fe_3_O_4_) nanoparticles. The measurements were nondestructive. Such a system can also be used for the detection of magnetic ink on old bills, which can result in false alarm signals in the detection of counterfeit notes using conventional magnetic ink testers.

**Figure 8:**
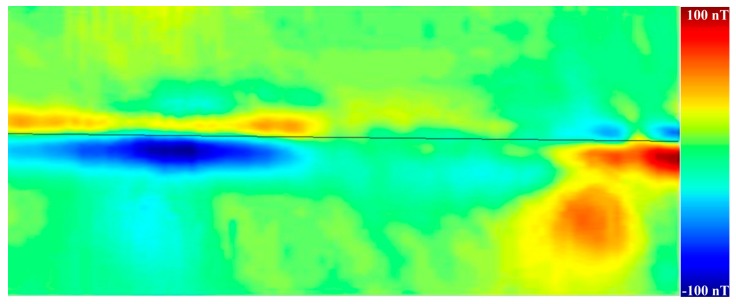
3D color-scale image of the magnetic field distribution measured over a weld seam (indicated by a black line) made by laser welding. The range of color-scale values is from −100 nT (blue) to 100 nT (red). The scanned area is 30 mm × 10 mm.

The nondestructive evaluation of magnetic features in stainless steel X5CrNi18-10 (German grade 1.4301, AISI 304) samples caused by welding and wear-out was performed. Although this corrosion-resisting austenitic steel is not magnetic, heat treatment or wear [53] partially transform non-magnetic austenite to ferromagnetic ά-martensite that is brittle and less resistant to corrosion. The detection of magnetic signals at weld seams provides valuable information about the quality of the welding. An example of a magnetic image of a weld seam made by laser welding of 1.4301 stainless steel plates is shown in Figure 8. The magnetic signal measured along such a weld seam is relatively weak compared to the more than 10 times stronger magnetic field above seams made using wolfram-inert-gas (WIG) welding of the same steel plates.

The wear-out of stainless steel plates was simulated by scratching [50] the plates using a diamond tip or engraving by a diamond drill. The measured magnetic signal originates from inclusions of the ferromagnetic ά-martensite form of the steel crystalline structure, which appear as a result of the plastic deformation of austenite in the contact area due to tribological stressing [53].

**Figure 9:**
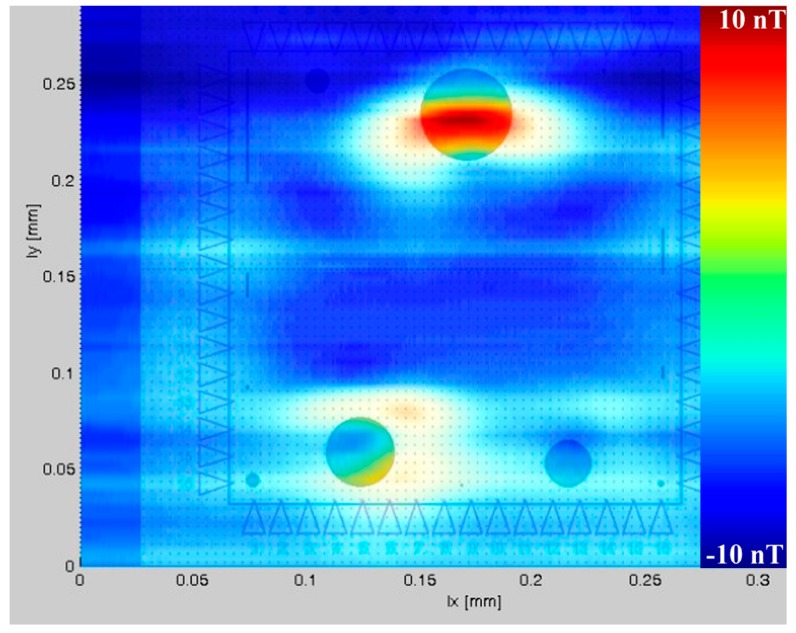
Magnetic field distribution of the demagnetized state of a 30-nm-thick Co film (contours showing the Co pattern have been added to the picture) prepared on a 50-nm-thick SiN membrane. The color scale represents magnetic fields of between −10 nT (blue) and 10 nT (red). Signals recorded from the magnetic domain structure of 40 µm, 30 µm and 20 µm dots are observable.

A SQUID microscope has been used for the investigation of the magnetization states of thin magnetic films and heterostructures intended for magneto-electronic devices and recording media. Bit patterns of information stored ferromagnetically on old floppy disks and hard disks have been evaluated. Changes in the distributions of magnetic stray fields in the Co/Al_2_O_3_/Co-tunnel junctions of tunneling magneto-resistive devices during their magnetization have been measured. The dependence of magnetic domain structure in thin Fe films on the thicknesses of (SiGe)_n_ barrier layers between them has been reported [54]. Magnetic stray fields originating from 30-nm-thick Co films fabricated using electron beam lithography on 50-nm-thick SiN membranes have been registered [50]. A measurement of the latter structure after demagnetization is shown in Figure 9. Measurements of stray magnetic fields using a SQUID microscope were performed in the frequency range 1–10 Hz and did not result in observable changes in magnetization.

The spatial resolution of the SQUID microscope of ~10 µm was limited primarily by the shape of the ferromagnetic tip of the magnetic flux antenna and the tip-to-sample separation. Additional sharpening of the tip by focused ion beam milling and the implementation of a tuning fork for controlling the tip-to-sample distance would improve the spatial resolution. The resulting thinning of the tip would deteriorate the magnetic field sensitivity. A possible solution involves optimization of the shape and material of the magnetic flux antenna. For example, Nanoperm M033 may result in better magnetic field sensitivity of the sensor [55].

**Figure 10:**
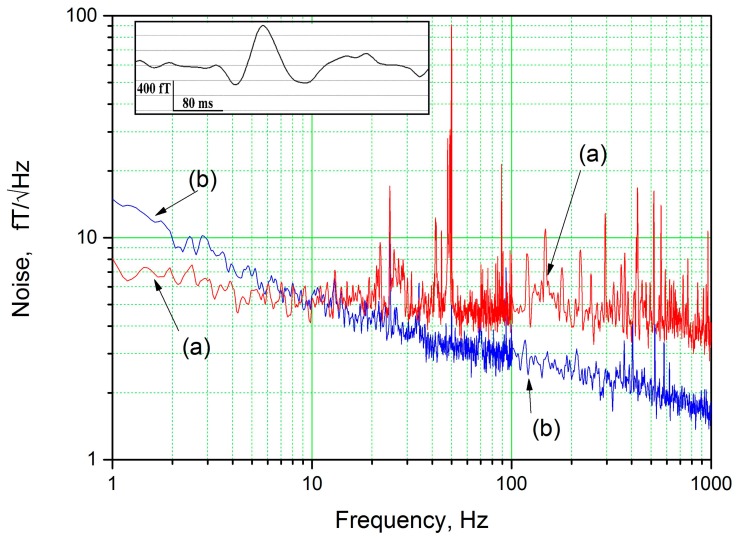
Noise spectra of a 20 mm high-T_c_ DC SQUID magnetometer measured at 77 K in a magnetic shield: (**a**) without a ferromagnetic antenna and (**b**) with a ferromagnetic antenna. The inset shows a measurement of human magnetoencephalography performed using a high-T_c_ DC SQUID magnetometer that has a sensitivity in the femto-Tesla range at low frequencies.

Replacement of the direct-coupled pick-up loop by a multilayer flux transformer improves transfer of the magnetic flux from the pick-up loop to the loop of the high-T_c_ DC SQUID (see [56] and references therein). For a 20-mm flip-chip magnetometric high-T_c_ SQUID sensor, a magnetic field resolution of ~4 fT/√Hz at 77 K was measured in magnetically shielded conditions [25,31]. This sensitivity was further improved to 2 fT/√Hz at 77 K by using an extremely soft magnetic flux antenna made from ferromagnetic Vitrovac 6025 foil [32]. In order to provide low values of Barkhausen and Johnson noise of the sensor, the magnetic flux antenna was assembled from ~250 pieces of 2-mm-wide 3.5-cm-long strips, which were cut in a direction perpendicular to the rolling direction of the foil and insulated on both sides by ~200-nm-thick insulating Al_2_O_3_ film. An example of noise measurement of the 20-mm flip-chip magnetometric high-T_c_ SQUID sensor with such a soft magnetic flux antenna in a magnetic shield is shown in Figure 10. The 20 mm sensors were initially developed for human magnetoencephalography [57] and other noninvasive noncontact investigations of biological objects. A composite ferromagnetic antenna can be prolonged through the walls of the cryostat in the future to measure the strongest component of the magnetic field in the nearest vicinity of the object under investigation. The combination of a superconducting flux transformer with a ferromagnetic flux antenna will also be useful for other NDE applications, such as improving the magnetic field resolution of a SQUID microscope or continuous non-invasive current monitoring of a high energy ion beam in a particle accelerator using a high-T_c_ SQUID sensor operating at temperature of up to 80 K.

Low-T_c_ DC SQUIDs with sizes of below 1 µm (“nanoSQUIDs”) have been fabricated on sharp tips of pulled quartz tubes and have demonstrated unprecedented spin sensitivities of ~0.38 μ_B_/√Hz [58] with spatial resolutions of ~20 nm [2]. The implementation of an electrically tunable multi-terminal SQUID configuration [59] provided optimal flux bias conditions by the direct injection of flux modulation and feedback current into the SQUID loop, thereby avoiding the need for the application of bias fields as high as ~0.4 T in the case of a 40-nm loop of a nanoSQUID. Such nanoSQUIDs can potentially be used for the nondestructive measurement of distributions of stray fields of magnetic nanoparticles and nanostructures, as well as for the nondestructive readout of the final states of superconducting flux qubits after their protection by sufficiently high potential barriers. The self-biasing of SQUIDs using YBCO-Nb JJs has also been realized [60]. NanoSQUIDs based on YBCO films and step-edge or bicrystal JJs should be able to operate at liquid nitrogen temperature or have a large *I_c_R_n_* product at lower temperatures [27,61].
